# Identification on Key Volatiles Contributed to Oviposition Preference of *Plodia interpunctella* (Hübner, 1813) (Lepidoptera: Pyralidae) from High and Normal Oleic Varieties of Peanut

**DOI:** 10.3390/insects15110866

**Published:** 2024-11-05

**Authors:** Chen Wang, Dianxuan Wang, Fangfang Zeng, Liang Chen, Xinxin Zhao, Xi Zhu, Junji Yao, Yihan Li

**Affiliations:** Grain Storage and Logistics National Engineering Research Center, National Grain Industry (Storage Insect Pest Control) Technology Innovation Center, Henan University of Technology, Zhengzhou 450001, China; 18638764239@163.com (C.W.);

**Keywords:** *Plodia interpunctella*, peanut variety, oviposition preference, volatile organic compound, electrophysiological response, behavior bioassay

## Abstract

The Indian meal moth, *Plodia interpunctella* (Hübner, 1813) (Lepidoptera: Pyralidae), a primary stored peanut insect pest, exhibited a significant difference in oviposition preference among normal-oleic peanuts (NOPs) and high-oleic peanuts (HOPs). Identifying key volatile organic compounds (VOCs) that are attractive or repellent to *P. interpunctella* is of great significance for the ecological management of pests. The profiles and contents of VOCs among NOP and HOP varieties were measured and compared, and key bioactive VOCs were further confirmed via an electroantennogram (EAG) analysis, as well as behavioral responses in Y-tube olfactometer and wind tunnel bioassays. Females prefer to lay eggs on NOP varieties more than on HOP ones. Acetophenone, nonanal, decanal, dodecane, 2,5-dimethylbenzaldehyde, and 4-ethyl-benzaldehy derived from tested peanuts elicited stronger antennal EAG responses. The results of the Y-tube olfactometer and wind tunnel bioassay showed that the relative high levels of nonanal, dodecane, and unique VOC acetophenone in NOP varieties have a significant attraction to *P. interpunctella*. Conversely, 4-ethyl-benzaldehyde and the unique VOC 2,5-dimethyl-benzaldehyde commonly found in HOP varieties exhibit notable repellent effects on *P. interpunctella*. These VOCs could contribute to the development of attractants or repellents derived from special peanut varieties for pest management.

## Simple Summary

The Indian meal moth, *Plodia interpunctella* (Hübner, 1813) (Lepidoptera: Pyralidae), exhibited significant differences in oviposition preference between normal-oleic peanuts (NOPs) and high-oleic peanuts (HOPs). In this study, we found key volatile organic compounds (VOCs) from NOP and HOP peanuts that were attractive or repellent to *P. interpunctella* females by gas chromatography/mass spectrometry (GC-MS), gas chromatography/electroantennographic detection (GC-EAD), and electroantennogram (EAG) analysis, as well as behavioral responses in Y-tube olfactometer and wind tunnel bioassays. The identification of key VOCs may provide a foundation for developing food-based attractants and repellents for pest control.

## 1. Introduction

The peanut, *Arachis hypogaea* (Linnaeus, 1753) (Fabales: Fabaceae), is an important oil and cash crop [1] and is widely distributed around the world. Peanut varieties are categorized as normal-oleic peanuts (NOPs) and high-oleic peanuts (HOPs) varieties based on the percentage of oleic acid oil. The proportion of oleic acid oil (18:1) in the total fatty acids of NOP is approximately within the range of 41–67%, whereas in HOP, it can be up to 80% [2]. HOP varieties have been widely promoted and commercially cultivated in many countries [3]. A series of HOP varieties have been developed in China, including the prominent varieties Kainong 301, Jihua 11, Jihua 13, Huayu 32, and so on, recently [4]. Peanuts are susceptible to storage insect infestations, such as *P. interpunctella*, *Ephestia cautella* (Walker, 1863) (Lepidoptera: Pyralidae), *Tribolium castaneum* (Herbst, 1797) (Coleoptera: Tenebrionidae), *Corcyra cephalonica* (Stainton, 1850) (Lepidoptera: Pyralidae), *Oryzaephilus surinamensis* (Linnaeus, 1767) (Coleoptera: Silvanidae), *Oryzaephilus mercator* (Fauvel, 1860) (Coleoptera: Silvanidae), *Rhyzopertha dominica* (Fabricius, 1792) (Coleoptera: Bostrichidae), and *Cryptolestes ferrugineus* (Stephens, 1831) (Coleoptera: Laemophloeidae) [5,6,7]. Among them, the Indian meal moth can cause serious damage to stored peanuts [8].

The larvae of the Indian meal moth feed on food and stored products, while adults can infest products by oviposition [9]. The moth evenly distributes its eggs based on a great many kinds and quantities of stored products [10]. Severe economic losses of stored grains and peanuts had been caused by this insect [11,12]. As an environmentally friendly strategy for pest management, attractant-baited traps have been used to monitor insect populations and capture insects in grain storage and food-processing facilities. Pheromone kairomone-baited dome traps and pheromone-baited flight traps effectively captured *male P. interpunctella* [13]. Food-based attractants have gained more attention and application due to their easy accessibility and low cost [14]. One female of this moth can lay 100–300 eggs in its lifespan and possesses a stronger flight performance [8]. By interfering with egg laying, targeting female attraction or repulsion based on food volatility could be further used for population control, either by attracting females into traps or expelling them from infected areas.

Insects can rely on volatile organic compounds (VOCs) to locate food [15], appropriate hosts [16,17], mating, oviposition sites [10], or to stay away from danger [18], and so on. Understanding insect behavior and identifying VOCs from foods may help develop attractants or repellents [19,20]. Hexanal, heptanal, and octanal collected from three kinds of dried fruits and whole-wheat flour attracted more *P. interpunctella* females to lay eggs in the screened net cages [19]. A mixture of VOCs ((E)-2-octenal, (E)-2-nonenal, and (E)-2-heptenal) from whole-wheat flour were oviposition attractants for mated females of *P. interpunctella*, but not for males and unmated females of the insect in pitfall olfactometers bioassay [21]. Inversely, VOCs 3-methyl-1-butanol from fungus-infected wheat was repellent to mated *P. interpunctella* females in the Y-tube olfactometer bioassay [22]. There is currently a lack of reports on typical VOCs emitted from peanuts that either attract or repel *P. interpunctella*.

According to our preliminary work, *P. interpunctella* females laid more eggs on peanuts compared to wheat, maize, and rice [23], and there were significant differences in oviposition preferences among 32 peanut varieties (including 25 NOP and 7 HOP varieties). We assume that the oviposition preference of *P. interpunctella* in different varieties of peanuts is mediated by some key VOCs derived from peanuts. The research aims to confirm this hypothesis and validate the key VOCs mediating oviposition preference or repelling in the tested peanut varieties. The purpose of the measurement is to (1) measure the oviposition preference of *P. interpunctella* females in the tested peanut varieties; (2) identify bioactive VOCs that trigger female antennal responses; and (3) verify individual active VOCs in female antenna and behavioral responses through electroantennography (EAG), Y-tube olfactometer, and wind tunnel bioassay. The research results can provide reference for the development of new attractants or repellents for the ecological management of this moth.

## 2. Material and Methods

### 2.1. Peanuts

The tested varieties of NOP (Yuhanghua 1, Yuhua 9326, Pukehua 5, Puhua 28, and Tianfu 3) and HOP (Jihua 13, Jihua 11, and Kainong 301) were kept at 4 °C in a freezer for one week after harvesting and drying at 42 °C for 48 h, provided by the Puyang Academy of Agricultural Sciences, Puyang, P R China. The shelled kernels were kept in a refrigerator at −15 °C two weeks for insect elimination. Then, the samples were kept in a room at 27 ± 1 °C 7 days before measurement. The chemical characteristics of each tested peanut were determined by proximate analysis [24], including proportions of protein, fat, and water (Table 1). The chemical characteristics of oleic acid and linoleic acid in peanuts were determined by using near-infrared spectroscopy (Table 1).

### 2.2. Insect Rearing

The population of *P. interpunctella* was collected from a grain depot located in Zhengzhou, P R China, and reared with diets (oats, broken corn, and yeast in a specific proportion of 19:19:2) for several generations at 27 ± 1 °C, 70 ± 5% r.h., and 16:8 (L:D) photoperiod. The selected larvae of females and males were kept in containers (5 cm in diameter and 3 cm in height) separately with diets until adult emergence in preparation. Male and female larvae were distinguished during the larval stage by a dark patch along the median plane of the mid-dorsal abdomen in males, formed by the testis, while females exhibited no such patch [25]. New emerged virgin male and female moths were coupled for oviposition preference assay. Two-day-old mated females were chosen for electrophysiological, Y-tube olfactometer, and wind tunnel bioassays [21].

### 2.3. Oviposition Preference Measurement

**Figure 1:**
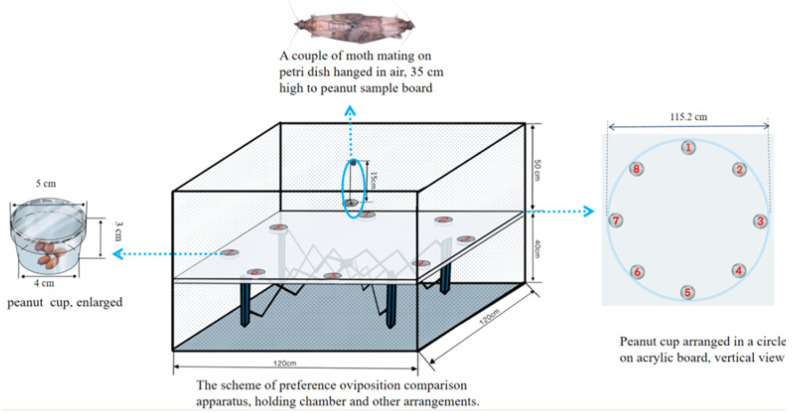
The scheme of the multiple-choice oviposition apparatus and material arrangement.

The oviposition preference measurement was conducted in a multiple-choice oviposition assay apparatus, which consisted of a cubical steel sheeting chamber, a peanut sample board containing eight peanut cups to hold peanuts, and a suspended petri dish (for mating moth preparation). The cup contained 20 ± 0.01 g of the kernel for one variety of peanut, which was set and distributed with other varieties in a circle along the margin of the board (Figure 1). A couple of mating moths were released into a petri dish hung under the lid of the apparatus for oviposition performance measurement. Seventy-two hours later, the cups with eggs and kernels were removed, and the egg number was checked under a stereomicroscope. Nine parallel experiments were carried out for each batch with eight varieties of peanut.

### 2.4. Volatile Organic Compounds Collection and Chemical Analysis

**Figure 2:**
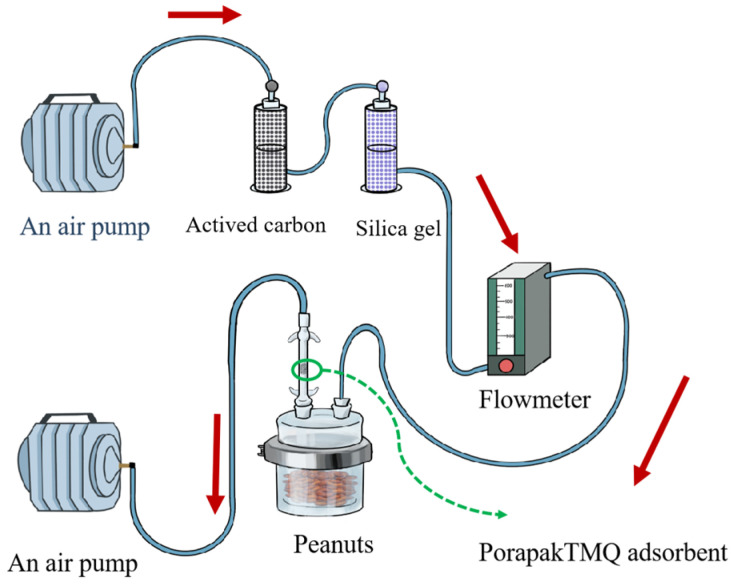
A schematic diagram of dynamic headspace collection systems for VOCs from peanuts.

The dynamic headspace sampling of VOCs was employed from tested varieties of peanuts. All sampling parts were connected with Teflon tubing. The dynamic headspace collection system is shown in Figure 2. An air pump (QC-1B, Institute of Labor Protection, Beijing, China) sequentially pulled an air stream through a vial containing activated carbon, a vial containing silica gel, a flowmeter, a glass jar (12.6 cm in diameter and 12.6 cm in height) containing peanut kernels, a VOC trap (0.9 cm in diameter, and 7 cm in height) filled with 100 mg PorapakTMQ adsorbent (Waters Company, Milford, MA, USA), and another air pump in same type (for airflow stream dewing out). The glass jar was cleaned with soap, washed with distilled water and acetone, and then heated at 40 °C for 6 h before collection/operation. The VOC trap was activated at 200 °C for 30 min before use. Four hundred grams of peanuts were sealed in a glass jar. The airflow was regulated at 300 mL/min by a flow meter. All dynamic headspace sampling parts without peanuts were run for 90 min for cleaning. The VOCs were sampled four times for each variety of peanut. Each collection was continued in a 12 h period. The air VOCs with no peanuts were collected as control. The VOCs were eluted from the adsorbent with 1 mL n-hexane (analytical grade, Aladdin, Shanghai, China), concentrated to 200 μL by a nitrogen evaporator (DN-36A, Shanghai Bilang Instrument Manufacturing Co., Ltd., Shanghai, China), and immediately stored at −80 °C. All measurements were carried out at 27 ± 1 °C, 70 ± 5% r.h., and a 16 L:8 D photoperiod.

The VOCs were analyzed by a QP2010 gas chromatography (GC)/mass spectrometer (Shimadzu Co., Ltd., Kyoto, Japan) equipped with a DB-5MS quartz capillary column (30 m × 0.25 mm × 0.25 μm; Agilent Technologies, Santa Clara, CA, USA). The collected samples (1 μL) were injected in splitless mode at a 250 °C injector temperature. Helium was used as carrier gas at 1.0 mL/min. The analytical conditions were as follows: The column oven was set to 35 °C for 3 min and then ramped up to 125 °C at 4 °C/min and held for 3 min. Subsequently, the temperature was increased to 165 °C at 4 °C/min and maintained for 3 min, and it was ultimately heated to 250 °C at 10 °C/min and remained for 5 min. In electron ionization mass spectra (EI) mode, the ionization voltage was 70 eV, the ion source temperature was 230 °C, the detector temperature was 250 °C, and the scan range of the mass spectra was 45–500 *m*/*z*. The VOCs were preliminarily identified by their mass spectra by comparing them using the NIST14.0 and Wiley 275 libraries. Positive identifications were confirmed when the spectra displayed a library match factor exceeding 90%. Further, the VOCs’ identification was made by comparing their retention index to those from the NIST-08 mass spectra library [26]. The retention indices were calculated based on the retention time of a sequence of alkanes C8–C40 for each selected VOC.

### 2.5. Chemicals

The tested chemicals, simulated with VOCs from the peanuts, included dodecane (99%), tetradecane (95%), hexadecane (98%), heptadecane (95%), decanal (95%) acetophenone (98%), 4-ethyl-benzaldehyde (98%), and 3,4-dimethyl acetophenone (98%); they were purchased from Aladdin Biochemical Technology Co., Ltd. (Shanghai, China). Nonanal (95%), 2,5-dimethyl-benzaldehyde (95%), and 2,4-di-tert-butylphenol (99%) were purchased from Merck, Inc. (Shanghai, China).

### 2.6. Electrophysiological Assays

**Figure 3:**
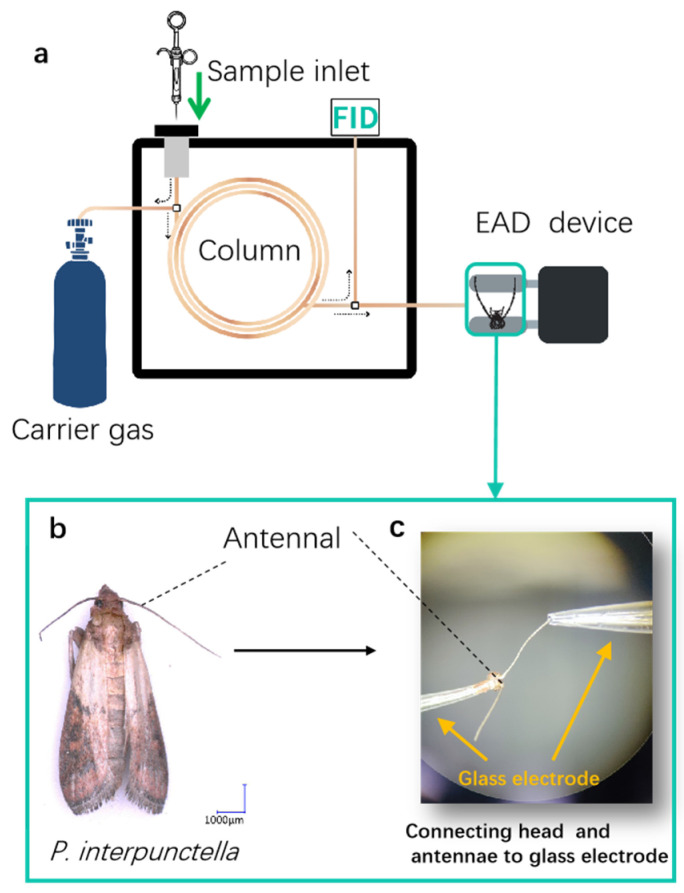
Identification of bioactive VOCs eliciting antennal responses in *Plodia interpunctella* females through gas chromatography/electroantennographic detection (GC-EAD). (**a**) A schematic representation of GC-EAD analysis. Dashed arrows indicate the flow of the carrier gas. (**b**) *Plodia interpunctella* female, from which the head (including antenna) was harvested. (**c**) A close-up view reveals the detailed arrangement of the head, antennal, and glass electrodes.

The NOP variety Yuhanghua 1, which exhibited the highest oviposition preference, and the HOP variety Jihua 11, which exhibited the lowest oviposition behavior to *P. interpunctella* females, were chosen for further analysis. The active VOCs emitted from the varieties Yuhanghua 1 and Jihua 11, which elicited antennal responses in the two-day-old mated females, were identified on a gas chromatograph (8860, Agilent Technologies, Santa Clara, CA, USA) with a quartz capillary column (DB-5MS, 30 m × 0.25 mm × 0.25 μm; Agilent Technologies, Santa Clara, CA, USA) equipped with an electroantennographic detector (EAD) (Syntech, Hilversum, Germany). The collected sample (1 μL) was injected (splitless mode), with the detector set at 250 °C. The high-purity helium (99.99%) served as the carrier, with a 1 mL/min fixed flow rate, for flame ionization detection (FID) and EAD. The column effluent was split in a 1:1 ratio between the FID and EAD. The column heating procedure was consistent with gas chromatography/mass spectrometry (GC-MS) analysis. The diagram combining the GC-EAD analysis is shown in Figure 3a. The female moth head preparation (with antennae) for EAG recordings was indicated in Figure 3b. The moth head was quickly cut off, and then antennae with cut head and (with a 1 mm tip removed) were inserted between two glass capillaries (length of 100 mm, outer diameter of 1.2 mm, and inter diameter of 0.9 mm; Sutter, CA, USA) filled with 0.1 mol/L NaCl solution. The head was inserted into a reference electrode, and the antenna tip was inserted into a recording electrode (Figure 3c). The antenna was placed 1 cm away from the air outlet. The recorded signal was amplified and converted by an IDAC-2 data-acquisition controller (Syntech, Hilversum, Germany), and the data acquisition for GC with EAD was analyzed by GC-EAD version 2014 v 1.2.5 software (Syntech, Hilversum, Germany). The VOC was confirmed if the antennal EAD peak matched the FID peaks of VOCs eluting from GC. The verification was conducted with five or more individual insects separately.

Acetophenone, dodecane, 2,4-di-tert-butylphenol, heptadecane, 4-ethyl-benzaldehyde, 2,5-dimethyl-benzaldehyde, 3,4-dimethyl-phenylethanone, tetradecane, and hexadecane, along with nonanal and decanal, which were more abundant in NOP than in HOP varieties, were dissolved in paraffin oil with a 1, 10, 50, and 100 μg/μL concentration, respectively. The antennae were connected following the method described in GC-EAD operations. After evaporation for 30 s, the tested chemicals (10 μL) were dripped onto a 0.43 cm^2^ filtered paper and put in a glass Pasteur pipette. The tip of the pipette was placed into a small hole in the wall of a glass tube, in which a steady airflow (300 mL/min) was delivered onto the prepared antenna. The antennae were delivered as a 0.5 s pulse, with an interval of 60 s between two consecutive stimulations. A solvent control (10 μL of paraffin oil) was tested before and after chemical stimulations. Each VOC was tested at 1, 10, 50, and 100 µg/µL of concentration, respectively. EAG recordings were obtained from six females one by one for the VOCs. The EAG response values of the controls were averaged before and after stimulation for each VOC assay. The EAG response values were calculated as *CT* − (*CK*_1_ + *CK*_2_)/2, where *CT* was the EAG amplitude of VOCs, and *CK*_1_ and *CK*_2_ were EAG responses to the first and the second control stimuli.

The EAG response was assayed to be stronger by a value ≥ 0.5 mV, moderate by 0.15 mV–0.5 mV, and weak by a value < 0.15 mV.

### 2.7. Y-Tube Olfactometer Bioassay

The selecting behavior response of the females to 11 VOCs was evaluated by a Y-tube olfactometer with an inner diameter of 4 cm, a common tube 30 cm in length, and two arms of 20 cm in length. The angle of the two arms was 75°. Each VOC was prepared in paraffin oil at a concentration of 50 μg/μL, and paraffin oil served as the control. The airflow was 300 mL/min; it passed through an activated carbon air filter, a bottle of distilled water, a flowmeter, and two odor containers; and it entered the olfactory arms finally. Them 10 μL of test solution was dropped onto a filter paper strip (3 cm × 0.5 cm) and placed in the odor container. The moth was recorded as making a choice if it crossed over 10 cm into one of two olfactory arms in 5 min and stayed for 10 s or more. The olfactory arms were cleaned with anhydrous ethanol and distilled water and dried at 150 °C for each group of five moths. A total of 60 moths were tested separately for each VOC. The bioassay was conducted under dark conditions at 27 ± 1 °C and 70 ± 5% r.h. The results of Y-tube olfactometer bioassays were expressed in terms of attractive rate and repellent rate, according to the following formula:

Attractive rate (%) = the number of females in the treatment arm/the number of females tested × 100%(1)

Repellent rate (%) = the number of females in the control arm/the number of females tested × 100%(2)

### 2.8. Wind Tunnel Bioassay

Nonanal, decanal, dodecane, hexadecane, acetophenone, 4-ethylbenzaldehyde, and 2,5-dimethyl-benzaldehyde (50 μg/μL), which evoked olfactory reactions in the females in the Y-tube olfactometer assay, were measured further by the wind tunnel test bioassay, with paraffin oil as the control. A wind tunnel (200 cm length × 60 cm height × 60 cm width) was used at 25 ± 2 °C, 70 ± 5% r.h., under scotophase, from 17:00 to 00:00 [27]. The filtered air was blown by a centrifugal fan and exited by another same type of fan, with an airflow of 50 cm/s. Two lamps (40 W, fitted with a red filter) were placed 10 cm above the tunnel, with an intensity of 10 lux for light condition. The rubber soaked with 20 μL of tested solution was placed in a petri dish (10 cm in diameter) affixed to a metal stand (15 cm in height), which was positioned 15 cm from the upwind end of the wind tunnel. A two-day-old mated female moth was placed in a glass tube (2.5 cm in diameter and 12.5 cm in height) affixed to another metal stand, 15 cm from the floor and 195 cm downwind from the odor sources. A female was released downwind into the wind tunnel and tested for 5 min. After each test, clean air was introduced into the wind tunnel for 15 min to eliminate any potential air pollution. The behavior responses of females to VOCs were recorded as follows: taking off; upwind flight (reaching 100 cm from the VOCs or halfway through the wind tunnel); approaching the source of VOCs (reaching 20 cm from the VOCs); and landing on the source of VOCs. Each treatment was carried out with 30 females, and each female was used only once. Three replications were performed for each treatment. The wind tunnel was cleaned with 75% alcohol and distilled water before each treatment.

### 2.9. Data Analysis

The data on egg number on tested peanuts, EAG response value among tested concentrations of each VOC, and the behavior response of female in the wind tunnel bioassay were analyzed by one-way analysis of Variance (ANOVA), followed by Duncan’s multiple range test (*p* < 0.05). The behavioral response of female moths to VOCs was analyzed via the *χ*^2^ test to examine the null hypothesis that the moths showed equal preference for the VOCs and the control. All statistical analyses were performed using IBM SPSS Statistics 26.0 (IBM Corp., Armonk, NY, USA).

## 3. Results

### 3.1. The Oviposition Preference of Plodia interpunctella Females

**Figure 4:**
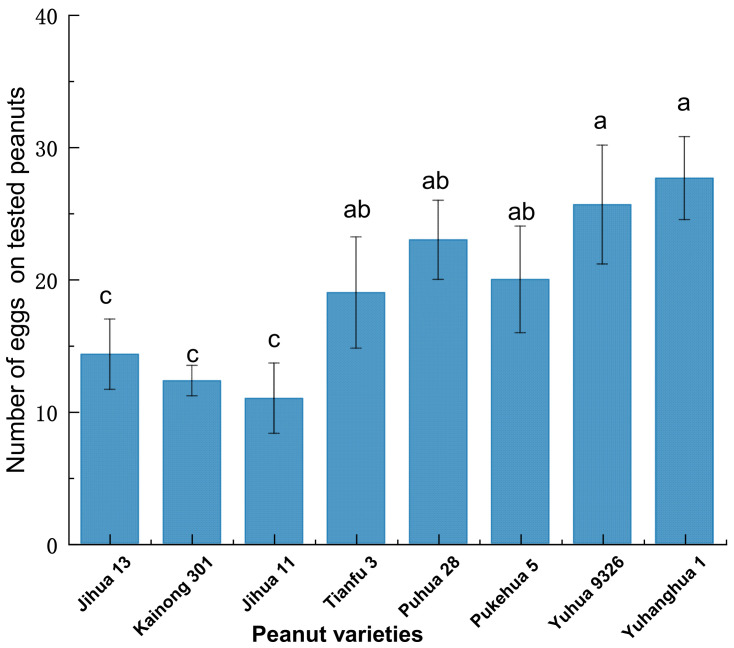
The oviposition preference comparison of *Plodia interpunctella* females among eight peanut varieties. Bars represent the number of eggs (mean ± SE) deposited by an individual female in 72 h. Distinct lowercase letters are employed to indicate statistically significant differences according to Duncan’s multiple range test (*p* < 0.05).

There was a significant difference in the number of eggs among the eight tested peanut varieties (F = 9.163; df = 7, 63; *p* < 0.001) (Figure 4), especially between NOP varieties (20–28 eggs) and HOP ones (9–12 eggs) (*p* < 0.05) (Figure 4). The order of egg production of peanut varieties from high to low is Yuhanghua 1 > Yuhua 9326 > Puhua 28 > Pukehua 5 > Tianfu 3 > Jihua 13 > Kainong 301 > Jihua 11. The NOP variety Yuhanghua 1 exhibited the highest preference for oviposition, with an egg production 3.1 times that of HOP Jihua 11.

### 3.2. Chemical Analysis

Forty-two VOCs from all of the tested varieties were analyzed via the GC-MS method, including 23 VOCs from Yuhanghua 1, 17 VOCs from Yuhua 9326, 10 VOCs from Puhua 28, 14 VOCs from Pukehua 5, 11 VOCs from Tianfu 3, 15 VOCs from Jihua 13, 16 VOCs from Kainong 301, and 20 VOCs from Jihua 11 (Table 2). These VOCs mainly included alkanes, aldehydes, and ketones, along with fewer types of alcohols, acids, and esters.

Dodecane was common to all NOP and HOP varieties. The relative content of dodecane in NOP varieties was higher than that in HOP varieties. Hexadecane was identified in all tested varieties, except for Pukehua 5, while 3,7-dimethyldecane was detected in all except for Jihua 13. Heptadecane and tetradecane were detected in most varieties, but heptane was not found in Pukehua 5 and Tianfu 3. Similarly, tetradecane was not found in the varieties Pukehua 5, Tianfu 3, and Jihua 13. Tridecane was identified in both Yuhua 9326 and Pukehua 5. The VOCs 5-methyl-5-propylnonane and 2-methyl hexacosane were measured in Yuhanghua 1, while decane was only detected in Pukehua 5.

Nonanal was the common VOC in all NOP and HOP varieties. In addition, decanal was absent from the NOP varieties Puhua 28 and Jihua 13. The relative contents of nonanal and decanal in NOP varieties were higher compared to those in HOP varieties. Benzaldehyde was uniquely identified in Pukehua 5, whereas 2,5-dimethyl-benzaldehyde was found in the HOP variety Jihua 11. The relative content of 4-ethyl-benzaldehyde in three HOP varieties was higher compared to that in the NOP variety Jihua 11 (*p* < 0.05).

The VOC 3,4-dimethyl-phenylethanone was identified in two NOP varieties, Yuhanghua 1 and Yuhua 9326, along with three HOP varieties. The relative content of 3,4-dimethyl-phenylethanone in three HOP varieties was higher compared to two NOP ones (*p* < 0.05). Acetophenone was detected in two NOP varieties, Yuhanghua 1 and Yuhua 9326. The VOC 6-methyl-5-hepten-2-one was identified in Pukehua 5.

The VOC 2,4-di-tert-butylphenol was identified in two NOP varieties, Yuhanghua 1 and Yuhua 9326, along with three HOP varieties. Additionally, 3-butyn-1-ol was identified in Tianfu 3 and Pukehua 5; 1-hexanol was detected in Puhua28 and Tianfu 3. The VOC 1-octen-3-ol was identified in Tianfu 3.

Hexanoic acid was identified in Pukehua 5 and Tianfu 3, while benzoic acid was uniquely detected in Tianfu 3. Additionally, butanedioic acid and diethyl ester were analyzed in the HOP variety Jihua 13.

### 3.3. Antennal Responses of the Females to Volatile Organic Compounds

**Figure 5:**
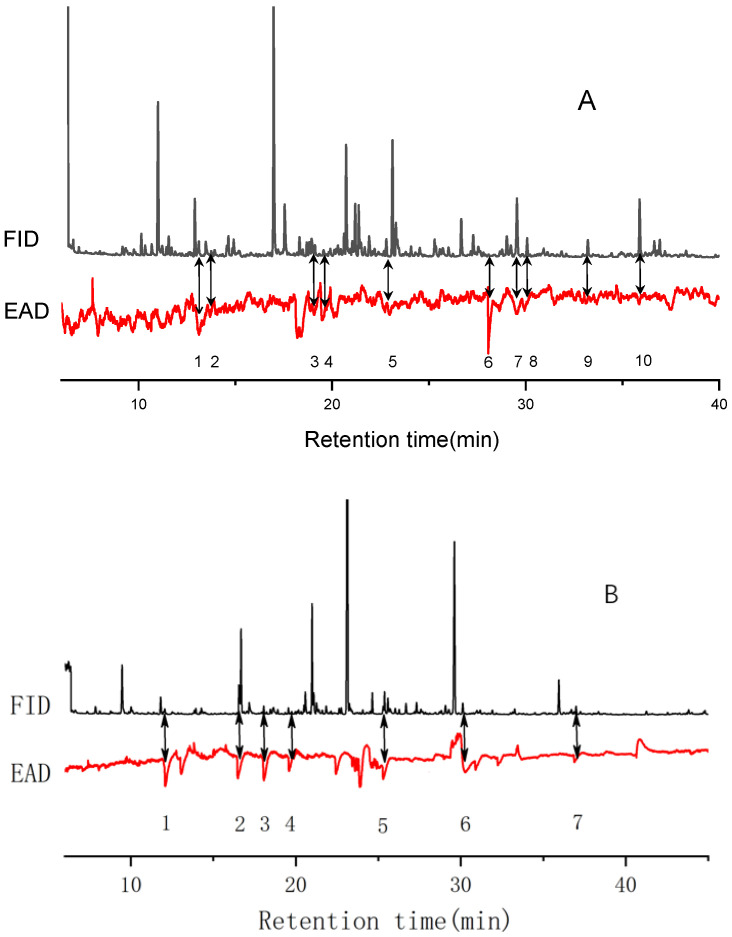
Responses of *P. interpunctella* females to Yuhanghua 1 (**A**) and Jihua11 (**B**) VOC detected by GC−EAD. In the panel A, the numbers of 1-10 represent the compounds of unidentified, acetophenone, dodecane, unidentified, tetradecane, unidentified, unidentified, 2,4-di-tert-butylphenol, hexadecane, unidentified, respectively; In the panel B, the numbers of 1-7 represent the compounds of 3,7-dimethyl-decane, 4-ethyl-benzaldehyde, 2,5-dimethyl-benzaldehyde, 3,4-dimethyl-phenylethanone, tetradecane, 2,4-di-tert-butylphenol, heptadecane, respectively.

Acetophenone, 3,7-dimethyl-decane, dodecane, 3,4-dimethyl phenylmethanone, hexadecane, heptadecane, tetradecane, 4-ethyl-benzaldehyde, 2,5-dimethyl-benzaldehyde, and 2,4-di-tert-butylphenol elicited the EAG responses in females (Figure 5).

**Figure 6:**
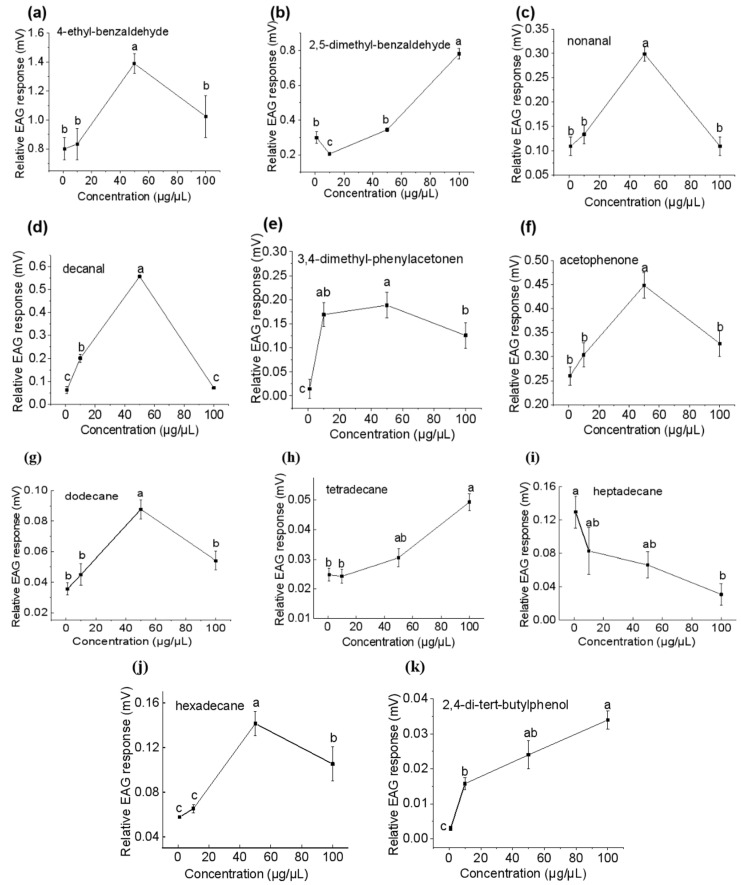
Comparative electroantennography responses (mean ± SE) of *Plodia interpunctella* females to volatile organic compounds at different concentrations (1, 10, 50, and 100 µg/µL): (**a**) 4-ethylbenzaldehyde; (**b**) 2,5-dimethyl benzaldehyde; (**c**) nonanal; (**d**) decanal; (**e**) 3,4-dimethyl phenylethanone; (**f**) acetophenone; (**g**) dodecane; (**h**) tetradecane; (**i**) heptadecane; (**j**) hexadecane; and (**k**) 2,4-di-tert-butylphenol. Different letters indicate significant differences (one-way ANOVA, Duncan’s multiple range test, *p* < 0.05).

As the VOC dose increased from 1 to 100 μg/μL, the EAG response of females to 2,4-di-tert-butylphenol, 2,5-dimethylbenzaldehyde, and tetradecane increased, while the responses to heptadecane decreased (Figure 6). The EAG responses of females to decanal, hexadecane, nonanal, 4-ethyl-benzaldehyde, 3,4-dimethyl-phenylethanone, acetophenone, and dodecane increased with concentrations and saturated at 50 μg/μL before decreasing (Figure 6). The highest EAG response was elicited by 50 μg/μL of 4-ethyl-benzaldehyde (EAG value of 1.39 mV, as noted below), 100 μg/μL of 2,5-dimethylbenzaldehyde (0.78 mV), 50 μg/μL of decanal (0.56 mV), and 50 μg/μL of acetophenone (0.45 mV), followed by nonanal, 3,4-dimethyl-phenylethanone, and hexadecane at 50 μg/μL, as well as heptadecane at 1 μg/μL (between 0.15 and 0.3 mV). The responses elicited by dodecane, tetradecane, and 2,4-di-tert-butylphenol are very weak.

### 3.4. Behavioral Responses

**Figure 7:**
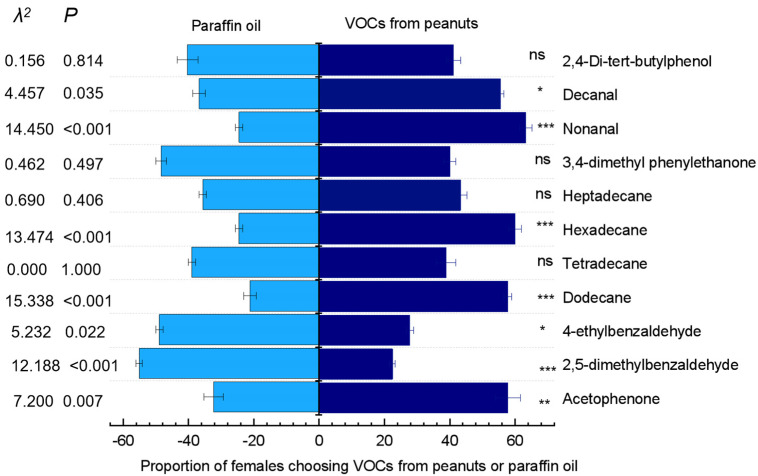
Behavioral responses of *Plodia interpunctella* females in the Y-tube olfactometer to test compounds at 50 µg/µL and the paraffin oil control. Thirty insects were tested per treatment. The dark blue bars on the right side and the light blue bars on the left side represent the responses to the test volatiles and paraffin oil, respectively. * *p* < 0.05; ** *p* < 0.01; *** *p* < 0.001; “ns” indicates no significant difference (*χ*^2^-test).

In the bioassay of Y-tube olfactometer, females were significantly more attracted to dodecane (*χ*^2^ = 15.338; *p* < 0.001), acetophenone (*χ*^2^ = 7.200; *p =* 0.007), hexadecane (*χ*^2^ = 13.474; *p* < 0.001), and nonanal (*χ*^2^ = 14.450; *p* < 0.001) than that to the paraffin oil control. The females had weaker attraction to decanal (*χ*^2^ = 4.457; *p* = 0.035), significant repulsion to 2,5-dimethyl-benzaldehyde (*χ*^2^ = 12.188; *p* < 0.001), and weak repulsion to 4-ethyl-benzaldehyde (*χ*^2^ = 5.232; *p* = 0.022). However, the females were neither attracted nor repelled by tetradecane, 2,4-di-tert-butylphenol (*χ*^2^ = 0.156; *p* = 0.814), heptadecane (*χ*^2^ = 0.690; *p* = 0.406), and 3,4-dimethyl phenylmethanone (*χ*^2^ = 0.462; *p* = 0.497) (Figure 7).

**Figure 8:**
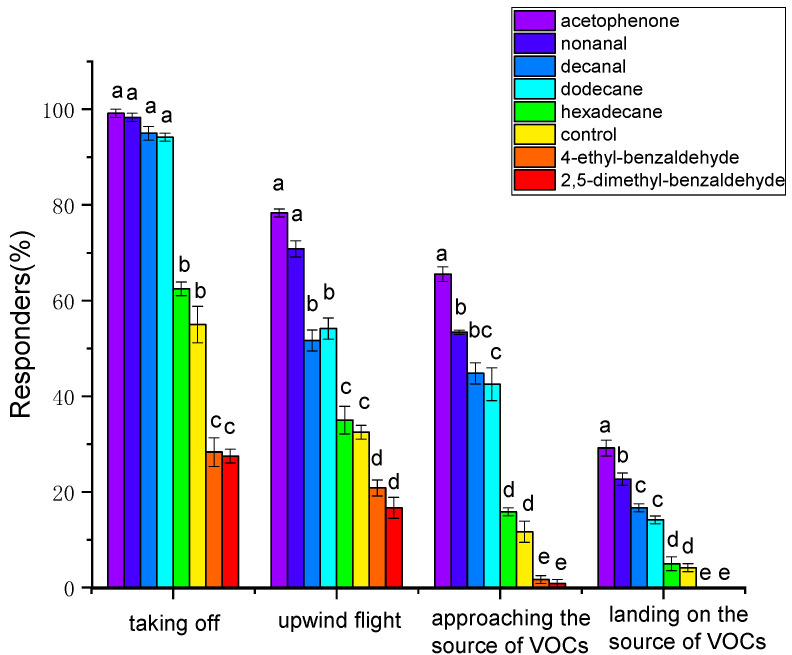
Behavioral response of *Plodia interpunctella* females towards 50 µg/µL of test volatile organic compounds in the wind tunnel. Different letters indicate significant differences among different volatile organic compounds (one-way ANOVA, Duncan’s multiple range test, *p* < 0.05).

In the wind tunnel bioassay (Figure 8), compared with the control group, acetophenone, nonanal, decanal, and dodecane significantly increased the proportion of female responding in all behavior responses, from taking off to landing on source of VOCs, while 2,5-dimethylbenzaldehyde and 4−ethylbenzaldehyde significantly reduced the proportion of female responding in all behavior responses (*p* < 0.05), and no significant difference was revealed in female responses to hexadecane (*p* > 0.05). Among reactive females, acetophenone elicited the highest proportion of all behavior responses, followed by nonanal, decanal, and then dodecane (*p* < 0.05). Acetophenone and nonanal attracted 29.16% and 22.67% of females to land on the VOC source, respectively, while neither 2,5−dimethylbenzaldehyde nor 4-ethylbenzaldehyde attracted any females to land on the VOC source.

## 4. Discussion

Grains and some foods can be either attractive or repellent to stored-product pests [28,29]. There is little attention paid to the oviposition preference of insects for storing peanuts in their preferences for certain storage products. Our valuable measurements demonstrate that compared to wheat, rice, and corn, some peanuts are significantly more attractive to *P. interpunctella* females [23]. We also found that *P. interpunctella* females had higher egg production in normal oleic peanut varieties than in high oleic ones. The larva and adults of *R. dominica* showed the strongest olfactory preference for wheat, followed by rice, maize, sorghum, and soybean [30]. Males and female maize weevil *Sitophilus zeamais* (Motschulsky, 1855) (Coleoptera: Curculionidae) were significantly attracted by maize and wheat seeds but were significantly repelled by alligator pepper, ginger, and black pepper [31]. The *Stegobium paniceum* (Linnaeus, 1758) (Coleoptera: Ptinidae) showed significant preferences for *Panax notoginseng* (Burkill, 1903) (Apiales: Araliaceae), followed by *Angelica sinensis* (Oliv., 1872) (Apiales: Apiaceae), *Gastrodia elata* (Blume, 1826) (Asparagales: Orchidaceae), and *Peucedanum praeruptorum* (Blume, 1826) (Asparagales: Orchidaceae) [28].

Insects can find and locate suitable food sources, mating, and ovipositing sites by VOCs [30,32]. The behavior responses of several stored grain pests to volatile organic compounds in grains was studied [33,34,35]. *P. interpunctella* females were attracted by 1-hexanol, phenylacetaldehyde, and 3-methyl-1-butanol, and (E)-2-nonenal from grains [21,22] and repelled by 4-oxoisophorone from the fungi-uninfected grain [22]. This study reports for the first time the behavioral response of *P. interpunctella* female to volatile organic compounds in different peanut varieties, which may provide valuable insights for the control of *P. interpunctella* in peanuts.

Active VOCs play an important role in the behavior response of insects and can be usually screened via the GC-EAD method [36,37]. Eleven special VOCs were selected through GC-EAD and GC-MS analyses in our treatments. There were significant differences in the number of compound kinds and relative content of VOCs among NOP and HOP varieties. Acetophenone was checked in NOP Yuhanghua 1 and Yuhua 9326 and 2,5-dimethyl-benzaldehyde only measured in HOP variety Jihua 11. The VOC 4-ethyl-benzaldehyde was more abundant in three HOP varieties, Kainong 301, Jihua 11, and Jihua 11, than in NOP variety Yuhua 9326. The relative content of dodecane and nonanal in three NOP varieties was higher than that in three HOP ones. The common VOCs, nonanal, dodecane, hexadecane, and decanal, from tested peanuts were also identified from wheat and rice [23]. The acetophenone was unique to NOP varieties and is considered to be a sweet odor; it has been reported in runner-type peanuts (*Arachis hypogaea* L., variety Georgia Green) [38] and may attract *P. interpunctella*.

Female moths exhibited strong EAG responses to 4-ethyl-benzaldehyde, 2,5-dimethyl-benzaldehyde, decanal, acetophenone, and nonanal. These results were similar to behaviors of *Oryzaephilus surinamensis* [39] and *Sitophilus oryzae* [40]. Y-tube and wind-tunnel assays revealed that dodecane, nonanal, decanal, and acetophenone were attractive to this moth, whereas 4-ethyl-benzaldehyde and 2,5-dimethyl-benzaldehyde had a repellent effect on females.

Nonanal and decanal from NOP and HOP varieties have been taken as attractants to *Callosobruchus maculatus* (F.) (Fabricius, 1775) (Coleoptera: Chrysomelidae) [41], *O. surinamensis* [39], *T. castaneum* [42], and *Sitotroga cerealella* (Olivier, 1795) (Lepidoptera: Gelechiidae) [43] in Y-tube olfactometer bioassays. Nonanal from whole-wheat flour attracted female moths of *P. interpunctella* for oviposition at low doses but had no attractive effect at high doses in Y-tube olfactometer bioassay [21]. Low concentrations of 1-hexanol and nonanal were attractive to mated female moths of *P. interpunctella* in the same bioassay [22]. Our results are consistent with the fact that the relative content of nonanal in NOP is higher than that in HOP ones (*p* < 0.05), and this fact may explain the significantly higher egg number of *P. interpunctella* on NOP varieties compared to HOP ones. Decanal was also a major component of the aggregation pheromone for the greater wax moth, *Galleria mellonella* (Linnaeus, 1758) (Lepidoptera: Pyralidae) [44]. Our result demonstrated decanal had a significant attraction to *P. interpunctella* females. Similarly, the relative content of decanal in NOP varieties is significantly higher than that in HOP ones (*p* < 0.05), which contributed to the oviposition preference of *P. interpunctella* females for NOP varieties.

Acetophenone was primarily detected in preferred oviposition stored products of *P. interpunctella*, such as wheat and dried fruits [45,46], and also measured in NOP varieties in this research here. In the tested NOP varieties, the unique VOC acetophenone appeared to have a synergistic effect with other VOCs, enhancing the oviposition preference of *P. interpunctella*. The VOC 4-ethyl-benzaldehyde, which repelled females, was detected to be significantly high level than that in three HOP varieties, Kainong 301, Jihua11, and Jihua 13, compared with NOP variety Yuhua 9326 (*p* < 0.05). The low oviposition preference in HOP varieties likely resulted from the high content of these deterrent VOCs. The unique 2,5-dimethyl-benzaldehyde, found in HOP Jihua 11 only, may synergistically interact with 4-ethyl-benzaldehyde to enhance its repellent effect on females. This combination of these VOCs likely played a significant role in the low oviposition number observed in Jihua 11. The VOC 3,4-diethylacetophenone, identified as having a high relative content in HOP and NOP varieties, despite eliciting strong electroantennographic (EAG) responses to the females, may not necessarily exert significant attractive effects.

## 5. Conclusions

Our results demonstrated that *P. interpunctella* females lay more eggs on NOP than on HOP varieties. The higher relative content of dodecane and nonanal, along with the unique presence of acetophenone in NOP varieties, showed effective attractiveness to females in Y-tube olfactometer and wind tunnel bioassays. In contrast, the higher relative levels of 4-ethyl-benzaldehyde and the exclusive presence of 2,5-dimethyl-benzaldehyde in HOP varieties acted as repellents for the females. The VOCs identified from NOP varieties could be utilized in the development of volatile-based attractants, while some VOCs originating from HOP varieties may serve as ingredients for repellents. This approach could play a crucial role in the integrated management of *P. interpunctella* for stored product protection. Certainly, future studies should further evaluate the field effectiveness of key VOCs in grain depots or food-processing facilities. Additionally, it is necessary to broaden the range of peanut varieties to provide insights into the diversity of VOC profiles and their impact on *P. interpunctella* and other stored product-insect behaviors based on these results.

## Figures and Tables

**Table 1:** The chemical characteristics of eight varieties of peanuts.

Peanuts Variety	Oleic Acid (%)	Linoleic Acid (%)	The Oleic/Linoleic Acid Ratio	Fat (%)	Protein (%)	Water (%)
Yuhua 9326	34.7 ± 1.9	35.7 ± 0.7	1.0 ± 0.1	49.3 ± 0.9	22.9 ± 0.8	7.1 ± 1.0
Tianfu 3	37.2 ± 2.7	38.5 ± 2.1	1.1 ± 0.1	46.9 ± 1.2	26.5 ± 0.9	6.8 ± 0.6
Pukehua 5	37.5 ± 3.1	39.3 ± 0.3	1.1 ± 0.1	54.6 ± 1.9	21.4 ± 1.0	7.0 ± 0.7
Puhua 28	37.7 ± 4.23	40.1 ± 1.0	1.2 ± 0.1	50.5 ± 1.0	23.4 ± 0.4	6.2 ± 0.5
Yuhanghua 1	39.2 ± 3.5	37.2 ± 0.4	1.2 ± 0.1	51.7 ± 0.3	21.8 ± 0.2	6.6 ± 0.2
Jihua 13	80.5 ± 0.3	3.2 ± 5.0	19.4 ± 0.6	52.8 ± 1.0	23.9 ± 0.7	6.8 ± 0.2
Kainong 301	80.5 ± 0.3	6.68 ± 2.6	17.5 ± 0.8	53.0 ± 0.7	21.0 ± 1.5	6.9 ± 0.1
Jihua 11	81.3 ± 0.3	3.4 ± 4.8	22.2 ± 13.5	53.3 ± 0.8	26.1 ± 1.8	6.9 ± 0.3

**Table 2:** Volatile organic compounds detected from eight varieties of peanuts by GC-MS method.

No.	VOCs	Retention Time	RI *^a^*	RI *^b^*	Relative Content (%)							
					Yuhanghua 1	Yuhua 9326	Puhua28	Pukehua 5	Tianfu 3	Jihua 13	Kainong 301	Jihua 11
1	3-butyn-1-ol	5.03	650	659	—	—	—	2.1 ± 0.6 a	4.2 ± 1.1 a	—	—	—
2	1-hexanol	5.23	849	860	—	—	0.1 ± 0.0 b		1.4 ± 0.1 a	—	—	—
3	benzaldehyde	7.54	968	982	—	—	—	0.4 ± 0.0	—	—	—	—
4	1-heptanol	8.91	946	960	—	—	—	0.21 ± 0.0	—	—	—	—
5	decane	8.94	1004	1015	—	—	—	5.3 ± 1.4	—	—	—	—
6	6-methyl-5-hepten-2-one	9.02	935	938	—	—	—	1.7 ± 0.2	—	—	—	—
7	1-ethyl-3-methylbenzene	9.16	984	964	0.1 ± 0.0	—	—	—	—	—	—	—
8	1-octen-3-ol	9.94	967	969	—	—	—	—	0.6 ± 0.0	—	—	—
9	octanal	9.93	1001	1005	—	—	2.0 ± 0.7 a	0.3 ± 0.1 b	—	—	—	—
10	limonene	10.04	1010	1018	—	—	—	1.2 ± 0.1	—	—	—	—
11	hexanoic acid	10.22	964	974	—	—	—	3.4 ± 1.0 b	5.4 ± 1.8 a	—	—	—
12	2,2,4,6,6—pentamethylheptane	10.14	1004	997	0.8 ± 0.0 a	—	—	—	—	—	—	—
13	4-methyldecane	11.54	1033	1059	0.3 ± 0.0 a	—	—	—	—	—	—	0.3 ± 0.0 a
14	3,7-dimethyl-decane	12.06	1043	1150	0.3 ± 0.1 f	0.6 ± 0.1 e	0.5 ± 0.0 e	2.1 ± 0.1 c	2.2 ± 1.0 b	—	1.0 ± 0.1 d	3.0 ± 0.5 a
15	undecane	12.91	1061	1154	0.3 ± 0.0 b	—	—	—	—	4.0 ± 0.6 a	—	0.5 ± 0.1 b
16	acetophenone	13.47	1072	1078	0.6 ± 0.2 a	0.2 ± 0.0 b	—	—	—	—	—	—
17	nonanal	14.91	1102	1102	0.5 ± 0.1 d	0.9 ± 0.1 c	0.4 ± 0.0 de	5.7 ± 0.3 a	1.5 ± 0.1 b	0.3 ± 0.4 de	0.2 ± 0.0 ef	0.3 ± 0.0 e
18	benzoic acid	17.03	1147	1150	—	—	—	—	1.4 ± 0.30 a	—	—	—
19	4-ethyl-benzaldehyde	16.55	1105	1108	—	2.7 ± 0.4 b	—	—	—	3.3 ± 0.2 b	25.3 ± 3.2 a	2.8 ± 0.1 b
20	2,5-dimethyl-benzaldehyde	18.06	1122	1137	—	—	—	—	—	—	—	0.9 ± 0.2 a
21	butanedioic acid, diethyl ester	18.09	1143	1151	—	—	—	—	—	1.5 ± 0.0	—	—
22	dodecane	18.31	1167	1214	1.8 ± 0.1 d	2.5 ± 0.1 c	9.1 ± 0.3 a	7.3 ± 0.3 b	1.6 ± 0.2 d	1.2 ± 0.1 e	0.9 ± 0.1 e	1.0 ± 0.1 e
23	decanal	18.69	1173	1161	0.3 ± 0.0 d	0.5 ± 0.0 c	—	1.8 ± 0.1 b	2.2 ± 0.1 a	—	0.1 ± 0.0 d	0.2 ± 0.0 d
24	4,6-dimethyl-undecane	18.82	1176	1193	0.9 ± 0.2	—	—	—	—	—	—	—
25	4-methyl-dodecane	18.91	1178	1249	1.1 ± 0.1 a	—	0.3 ± 0.1 c	—	—	0.5 ± 0.1 b	—	0.45 ± 0.03 b
26	2,4-dimethyl-benzaldehyde	18.94	1179	1180	0.5 ± 0.2 a	0.7 ± 0.1 a	—	—	—	—	—	—
27	isophthalaldehyde	19.38	1210	1284	—	0.3 ± 0.0 a	—	—	—	—	—	—
28	tridecane	20.64	1302	1313	—	0.7 ± 0.1 a	—	2.6 ± 0.5 a	—	—	—	—
29	5-methyl tetradecane	20.62	1213	1454	0.3 ± 0.0 c	—	—	—	—	1.0 ± 0.1 a	0.7 ± 0.1 b	0.8 ± 0.2 b
30	3,4-dimethyl-phenylethanone	20.72	1215	1255	3.0 ± 0.2 d	5.1 ± 0.1 c	—	—	—	8.4 ± 0.6 ab	10.2 ± 0.9 a	9.4 ± 0.5 ab
31	4-ethylphenyl-ethanone	21.37	1231	1242	1.5 ± 0.3 c	—	—	—	—	4.6 ± 0.1 b	7.6 ± 0.9 a	—
32	5-methy-5-propylnonane	21.49	1233	1229	0.1 ± 0.0	—	—	—	—	—	—	—
33	4,6-dimethyl-dodecane	22.80	1264	1285	0.2 ± 0.0 d	0.8 ± 0.1 c	1.0 ± 0.1 bc	—	—	2.7 ± 0.4 a	1.3 ± 0.1 b	1.3 ± 0.1 b
34	tetradecane	25.29	1383	1413	0.3 ± 0.0 d	0.6 ± 0.1 b	0.5 ± 0.0 c	—	—	—	1.0 ± 0.1 a	0.4 ± 0.1 cd
35	1,1′-(1,4-phenylene)bisethanone	26.67	1400	1378	0.4 ± 0.2 e	3.2 ± 0.1 b	—	—	—	4.4 ± 0.1 a	1.9 ± 0.1 d	2.3 ± 0.1 c
36	2,4-di-tert-butylphenol	30.07	1455	1494	0.7 ± 0.1 c	2.1 ± 0.1 b	—	—	—	3.2 ± 0.3 a	2.1 ± 0.1 b	0.7 ± 0.1 c
37	hexadecane	33.21	1560	1612	0.7 ± 0.1 d	0.5 ± 0.1 d	11.1 ± 1.3 a	—	1.7 ± 0.2 bc	1.2 ± 0.1 cd	0.5 ± 0.1 d	2.3 ± 0.2 b
38	heptadecane	36.93	1685	1711	1.0 ± 0.1 c	0.4 ± 0.1 e	0.7 ± 0.1 d	—	—	2.3 ± 0.1 b	2.4 ± 0.1 a	0.5 ± 0.1 e
39	eicosane	37.02	2002	2009	0.3 ± 0.0 e	0.4 ± 0.1 d	—	—	—	2.6 ± 0.1 a	1.5 ± 0.1 b	1.0 ± 0.1 c
40	dibutyl phthalate	44.80	2168	2037	0.1 ± 0.0 d	—	—	—	0.4 ± 0.1 c	—	1.2 ± 0.2 a	0.9 ± 0.2 b
41	2-methyl-hexacosane	51.43	2617	2656	0.1 ± 0.0	—	—	—	—	—	—	—
42	phthalic acid, di(2-propylpentyl) ester	52.23	2689	2704	—	—	—	0.1 ± 0.0 b	—	—	—	0.6 ± 0.1 a

Data in the table are shown as mean ± SE (N = 3), and different letters in the same row indicate a significant difference (one-way ANOVA, Duncan’s multiple range test, *p* < 0.05); “—” means that the volatile organic compounds are not detected in extracts of peanuts. *^a^* Retention index was determined on a DB-5 MS column using a homologous series of n-alkanes (C8–C40). *^b^* Retention index was obtained from the literature for compound whose identity was established based on comparison of retention time and mass spectra data with authentic standard.

## Data Availability

The data presented in this study are available upon request from the corresponding authors.
